# Trends in Operative Delivery in Tikur Anbessa Specialized Hospital, Addis Ababa, Ethiopia: A 5 years' Retrospective Review

**DOI:** 10.4314/ejhs.v31i6.15

**Published:** 2021-11

**Authors:** Yeshiwas Abebaw, Eskinder Kebede

**Affiliations:** 1 Department of Obstetrics and Gynecology, School of Medicine, CHS, Gondar University, Gondar, Ethiopia; 2 Department of Obstetrics and Gynecology, School of Medicine, CHS, Addis Ababa University, Addis Ababa, Ethiopia

**Keywords:** Operative vaginal delivery, vacuum, forceps

## Abstract

**Background:**

Operative vaginal delivery refers to the use of measures to accomplish vaginal delivery through the use of instruments, mainly obstetric forceps and vacuum cups. In developed countries, the rate of cesarean section is increasing for fear of vaginal delivery complications, including instrumental delivery. This study was done to explore trends of operative vaginal deliveries and their characteristics

**Methods:**

A cross-sectional, facility-based retrospective study was conducted over a period of five years July 1, 2011, to June 30, 2016, using data collected from the labor ward logbook, patient charts. Data were coded, entered, using SPSS version 20 statistical software. Descriptive statistical analysis was used to describe and analyze the data into graphs and tables

**Results:**

The rates of operative vaginal delivery and cesarean section over the five-year study period were 11.9% and 30.4%, respectively. The trend in the operative vaginal delivery rate declined from 15.8% in July 2011 to 9.9% in June 2016, while it shows a noticeable rise in cesarean section rate 25.4% to 33.8%. The trend in the use of vacuum has shown a sharp decrease from 58% in the 1st year to 10.5% in the fifth year of the study period. There is a rise in the use of forceps from 42% of all operative vaginal delivery in the first year to 89.5% in the fifth year

**Conclusion:**

This study shows that the rate of operative vaginal delivery has declined. The use of vacuum-assisted delivery has especially decreased compared to that of forceps-assisted delivery.

## Introduction

Operative vaginal delivery, during which the second stage of labor is shortened with the assistance of a vacuum device or forceps, is an age-long obstetric practice used to expedite delivery or avert recourse to cesarean delivery (1). It is a vital component of basic emergency obstetric care worldwide and remains an integral part of obstetrician's duties. It may take the form of instrumental deliveries, employing obstetric forceps and vacuum extractor or operative procedures like symphysiotomy or destructive operations performed to achieve vaginal delivery in dystocia with or without a living fetus (2).

Operative vaginal delivery is an important management option in clinical situations where delivery needs to be expedited. In the setting of indications such as maternal exhaustion, a concerning fetal heart rate tracing, or maternal medical benefit from shortening second stage, operative vaginal delivery represents an alternative to cesarean delivery (3).

Resident in the use of both obstetric forceps and forceps deliveries is experiencing a precipitous decline in the United States. Current minimum training requirements are insufficient to ensure competency in this skill. These trends bear striking similar to observations regarding the decline and ultimate extinction of biologic species and portend the inevitable disappearance of this valuable skill from the obstetric armamentarium (4).

Most women wish to have a spontaneous vaginal delivery. Yet in the countries, (Scotland, Ireland, Canada, Australia, England, and the USA) 1 in 3 women will face an operative delivery and 1 in 9 an assisted vaginal delivery (3). The incidence of operative vaginal delivery in developed countries ranges from 10 – 15% in the UK to 4.5% in the USA (3).

According to the birth certificate data from the National Vital Statistics Report, forceps or vacuum-assisted vaginal delivery was used for 3.6% of births in the United States in 2010 (5), and it accounted for around 11% and 17.3% of births in the Royal College of Obstetricians and Gynecologists, Australia (6), and in Tikur Anbessa Specialized Hospital, (7) Ethiopia, respectively.

The declining trend of instrumented deliveries in the second stage of labor contrasted with an increasing number of CSs. It continues to fuel the perception that instruments are potentially harmful to fetuses and mothers and that cesarean delivery is preferable in most circumstances. The decrease in the number of physicians with the training and required skill to perform these procedures as well as the fear of litigation compounds, the problem of declining instrumental use even further (3), (8), (9). In Sub-Sahara, an operative vaginal delivery rate of < 1% has been reported (10).

The various rates of operative vaginal deliveries (OVDs) and CSs suggest that the approach to similar clinical situations is radically different among specialists of different latitudes, resulting in different behaviors and obstetric interventions. These national differences have also been observed in different regions of several countries (10).

The information available is of insufficient quality to recommend one instrument over the other in each specific case. Given an indication for OVD, the choice of instruments depends on the particular indication for the procedure, the anesthesia (in place or available), the availability of instruments, the training and experience of the obstetrician, and the preference of the patient and physician (3). A meta-analysis of studies comparing the use of vacuum and forceps has suggested that vacuum delivery is the method of choice for instrumented delivery (8).

The national cesarean section rate of Ethiopia increased from 0.7% in 2000 to 1.9% in 2016, with an increase across seven of the eleven administrative regions of the country. Addis Ababa had the highest cesarean section rate (21.4%) in 2016 and the greatest increase since 2000 (11). In the adjusted analysis, women who gave birth in a private health facility had a 78.0% higher incidence of cesarean section (adjusted prevalence ratio (aPR) (95% CI) 1.78 (1.22, 2.58)) compared with those giving birth in the public health facility (11).

At Mettu Karl Hospital, Oromia Regional State, southwest Ethiopia, out of the total 3346 deliveries during the study period, there were 984 (29.4%) operative deliveries. Cesarean section were 727 (73.9%), vacuum 190 (21.7%), Forceps 45 (1.3%) and destructive delivery 22(0.65%) (12).

In developing countries such as Ethiopia, where CS is not as freely available and institutional delivery is very low when an operative intervention is required in the second stage of labor, the options, risks, and benefits of vacuum, forceps, and CS must be considered and weighed up. Therefore, an instrumented vaginal delivery deserves as much consideration, thought, and preparation as an alternative to an abdominal delivery (3).

The OVD rate varies greatly between settings, the Ideal rate is unknown, but is said to be underused.

This study will determine the trends in operative delivery rates. Studies have shown that vacuum is the instrument of choice. However, the practice may differ. This study will identify the pattern in the proportion of instruments used to assist vaginal delivery in the 2^nd^ stage of labor at the Tikur Anbessa Hospital over five years.

## Methods and Material

The study aimed to describe the trends in operative delivery rates over time and determine associated factors.

An Institution-based retrospective cross-sectional study was conducted in Tikur Anbessa Specialized Hospital (TASH), one of the tertiary and specialized hospitals in Addis Ababa, the capital city of Ethiopia. The hospital has a comprehensive antepartum, intrapartum, and postpartum care, including neonatal intensive care unit. It serves as a referral center for all hospitals in the country. It has an average monthly delivery of 200–300 and is staffed with gynecologists and obstetricians, residents, midwives, and clinical nurses. It is purposely selected for this study assuming that it is staffed by clinicians capable of doing instrumental delivery. The study was conducted over five years from July 1, 2011, to June 30, 2016.

All women delivered by the instrument during the study period were included. Data were collected using a semi-structured questionnaire-form the mothers' and their neonates' charts, labor ward reporting logbooks, and instrumental delivery formats. Data were collected by the principal investigator and three junior residents. The completeness of the data collected was checked every day.

Ethical clearance was obtained from the Research and Publication Committee of the Department of Gynecology and Obstetrics, Addis Ababa University. Communication with the medical director of the hospital was made through formal letters obtained from the Faculty of Medicine, Addis Ababa University, and permission was obtained from the medical director to use the charts of mothers and their neonates.

Data were coded, entered, and cleaned using SPSS version 20 statistical software. Descriptive statistical analysis was used to describe and analyze the data into graphs and tables for easy interpretation. The analysis focused on the trends of OVD, distribution of VAD and FAD, indication for OVD, and station at the application of instruments were assessed. Results were expressed using tables, graphs, and percentages.

**Operational definition**: Operative vaginal delivery: Is when the second stage of labor is shortened with the assistance of a vacuum device or forceps

## Results

There were 12,995 deliveries at TASH during the five-year study period. A review of the delivery log, maternal card, and instrumental delivery formats revealed 1,547 instrumental vaginal deliveries (11.9% of total deliveries) and 3,946 cesarean deliveries (30.4%). Of the 1,547 instrumental deliveries, 1,342 (86.7%) cases were retrieved, included are 915 (68.2%) forceps and 427 (31.8%) vacuum-assisted vaginal deliveries. The trends in operative vaginal delivery (OVD) rates over the five-year study period (July 1, 2011, to June 30, 2016) were shown to be declining, from 15.8% in the period between 01/07/2011 and 30/06/2012 (the 1^st^ year of the study period). In the subsequent years of the study period, rates of operative vaginal delivery were 13.2%, 11.4%, 9.8%, and 9.9%, respectively (Table 1).

**Table 1:** Distribution of the number and rates of OVD and CS over the five-year study period July (2011 to June 2016)

	OVD		CS		SVD		Total Deliveries

Period	No.	%	No.	%	No.	%	
**Year 1 (2011)**	416	15.80%	667	25.40%	1541	58.70%	2624
**Year 2 (2012)**	276	13.20%	509	24.30%	1310	62.50%	2095
**Year 3 (2013)**	277	11.40%	776	31.90%	1381	56.70%	2434
**Year 4 (2014)**	296	9.80%	1033	34.40%	1671	55.70%	3000
**Year 5 (2015)**	282	9.90%	961	33.80%	1599	56.30%	2842
**Total**	**1547**	**11.90%**	**3946**	**30.40%**	**7502**	**57.70%**	**12,995**

The trends in the type of instrument used to assist delivery; the use of vacuum showed a sharp decrease from 58% of all OVD in the 1^st^ year of the study period to 10.5% in the fifth year of the study period. In the subsequent years of the study period, rates of vacuum delivery were 43.8%, 29.8%, and 9.9%, respectively.

**Figure 1:**
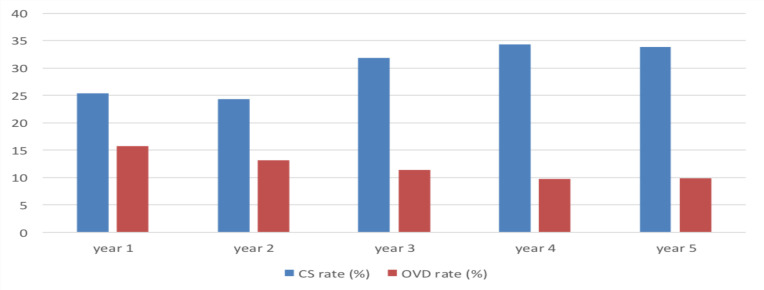
OVD rates Vs. CS rates over the five-year study period

**Figure 2:**
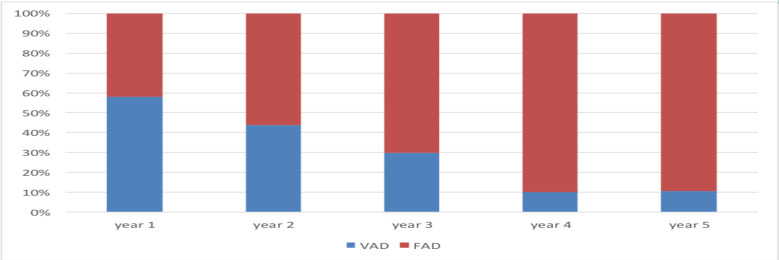
Proportion of VAD and FAD of total OVD over the five-year study period

On the other hand, there is a rise in the use of forceps from 42% of all OVD in the first year to 89.5% in the fifth year. (Table 2, Figures 1 and Figure 2). The trends in cesarean delivery over the five study periods (shares) were shown to have net increasing rates from 25.4% to 31.9% to be 25.4%, 24.3%, 31.9%, 34.4%, and 33.8%, respectively.

**Table 2:** Frequency and percentage distribution of VAD and FAD over the five-year study period (July 2011 to June 2016)

Type of OVD	VAD		FAD		Total

Period of OVD	N (%)	Rate	N (%)	Rate	
**Year 1**	199 (58.0)	7.58%	144 (42.0)	5.49%	343
**Year 2**	102 (43.8)	4.87%	131 (56.2)	6.25%	233
**Year 3**	73 (29.8)	3.00%	172 (70.2)	7.07%	245
**Year 4**	27 (9.9)	0.90%	247 (90.1)	8.23%	274
**Year 5**	26 (10.5)	0.92%	221 (89.5)	7.78%	247
**Total**	427 (31.8)	3.29%	915 (68.2)	7.04%	1343

The demographic characteristics of the maternal age were ranged between 15 and 40 years. The mean age was 25.9 (+ 4.5) for those delivered by vacuum and 25.6 (+ 4.5) for forceps deliveries. Primipara accounts for 66.3% of vacuum and 71.7% of forceps deliveries. The gestational age based on LNMP was unknown in 31.1% of vacuum and 28.4% of forceps deliveries. Among those with known GA, 261 (61.1%) of VAD (Vacuum Assisted Delivery) and 549 (60%) of FAD (Forceps Assisted Delivery) were term, GA (Gestational Age) was post-term in 32 (7.5%) of VAD and 72 (7.9%) of FAD, and preterm in one case of VAD and 34 (3.7%) of FAD.

When considering indications for the application of operative vaginal deliveries, fetal distress is the most frequent indication, accounting for 48%, followed by prolonged second stage; maternal cardiac illness and hypertensive disorder of pregnancy each account for 34.2%, 11.9%, and 5.6%, respectively. Forceps are the more frequently used instruments in all listed indications in both primipara and multiparas. (Tables 3 and 4)

**Table 3:** Frequency distribution of VAD and FAD by maternal age, parity, gestational age, and birth weight (July 2011 to June 2016) (VAD =427, FAD =915)

	VAD		FAD		
	
	No.	Percent	No.	Percent	
	**Maternal age (years)**				
**15 – 19**	23	5.4	50	5.5	73
**20 – 24**	142	33.3	338	36.9	480
**25 – 29**	161	37.7	358	39.1	519
**30 – 34**	76	17.8	126	13.8	202
**35 +**	25	5.9	43	4.7	68
**Total**	**427**	**100**	**915**	**100.0**	**1342**
	**Parity**				
**1**	283	66.3	656	71.7	939
**2 – 4**	137	32.1	255	27.9	392
**>4**	7	1.6	4	0.4	11
**Total**	**427**	**100**	**915**	**100**	**1342**
	**Gestational age (weeks)**				
**Unknown**	133	31.1	260	28.4	393
**< 37**	1	0.2	34	3.7	35
**37 – 41 + 6**	261	61.1	549	60	810
**> 42**	32	7.5	72	7.9	104
**Total**	**427**	**100**	**915**	**100**	1342
	**Birth weight (grams)**				
**<2500**	20	4.7	103	11.3	123
**2500 – 3999**	393	92	791	86.4	1184
**>4000**	14	3.3	21	2.3	35
**Total**	**427**	**100**	**915**	**100**	**1342**

**Table 4:** Frequency distribution of VAD and FAD by indication for OVD and station at the application of instruments (July 2011 to June 2016)

Indication	VAD		FAD		Total	

	No	%	No	%	No	*% Total*
***Fetal distress***	183	42.9	456	49.8	639	47.6
***Prolonged 2^nd^ stage***	201	47.1	253	27.7	454	33.8
***Maternal cardiac disease***	22	5.2	136	14.9	158	11.8
***Hypertensive disorder***	14	3.3	61	6.7	75	5.6
***Previous c/s***	2	0.5	3	0.3	5	0.4
***others***	5	1.2	6	0.7	11	0.8
Total	427	100.0	915	100.0	1342	100.0
***Station at application***						
***Station***						
***Outlet***	177	41.5	553	60.4	730	54.4
***Low***	201	47.1	359	39.2	560	41.7
***Mid-cavity***	49	11.5	3	0.3	52	3.9
**Total**	427	100	915	100	1342	100.0

Of the 427 VAD, 127 (41%), 200 (47.4%), and 49 (11.6%) were outlet, low, and mid cavity applications, respectively while for the 915 FAD, it was 552 (60.4%), 359 (39.3%), and 3 (0.3%), respectively. Of all the outlets' instrument applications, 23.9% were vacuum, while 35.8% of low and 94.2% of mid-cavity applications were vacuum. Among the instrumental deliveries, 93% were in the occipito anterior position, while 27 (2%), 65 (4.8%), and 2 (0.1%) were delivered in the occipito posterior, occipito anterior, and mento-anterior positions.

## Discussion

Even although operative vaginal delivery is one of the key elements of essential obstetric care, its role is undervalued. The incidence of operative vaginal delivery in this study was 11.9% (7.1% FAD vs. 3.3% VAD rate) and cesarean delivery rate of 30.8%. The rate of operative vaginal delivery decreased overtime when these results were compared to those of a study done in 2000GC (13) at TASH, which showed 18.5% (13.8% FAD vs. 4.7% VAD rate) while reporting a comparable cesarean rate of 27.8%. The OVD rate in developed countries ranges from 10 – 15% in the UK to 4.5% in the USA, and the CS rate are at 31.8% (2007) (3, 8, 14).

The OVD rate varies greatly between settings, with the ideal rate being unknown or underused. In Sub-Saharan countries, an operative vaginal delivery rate of < 1% has been reported. The various rates of operative vaginal deliveries (OVDs) and CSs suggest that the approach to similar clinical situations is radically different among specialists of different latitudes, resulting in different behaviors and obstetric interventions. These national disparities have also been observed within different regions of several countries (3, 10).

The study also revealed a decline in the operative vaginal delivery rate and rise of cesarean delivery over the five-year study from 15.8% OVD rate and 25.4% CS rate in the first year of the study period, (July 1, 2011, to June 30, 2016) to 13.2%, 11.4%, 9.8%, and 9.9% OVD rate and 24.3%, 31.9%, 34.4% and 33.8% CS rates in the subsequent four years of the study period, respectively. The pattern in the type of instrument used to assist vaginal delivery has been shown to shift from vacuum to forceps. The proportion of VAD was 58% in 2011 but progressively decreased to 43.8%, 29.8%, 9.9%, and 10.5% in the subsequent years. Vacuum-assisted delivery accounted for 31.8% of all OVD over the five year study period.

Although frequently used interchangeably, forceps and vacuum denote both a rather large group of mechanically dissimilar instruments and the procedure of delivering fetuses with them. Experts often provide conflicting evidence for and against the use of these procedures (3),(14).

The information available locally is of insufficient quality to recommend one instrument over the other in each specific case. Given an indication for OVD, the choice of instruments depends on the particular indication for the procedure, the anesthesia (in place or available), the availability of instruments, the training and experience of the obstetrician, and the preference of the patient (3).

The worldwide declining trend of instrumented deliveries in the second stage of labor contrasted with an increasing number of CSs continues to fuel the perception that instruments are potentially harmful to fetuses and mothers and that cesarean delivery is preferable in most circumstances. The decrease in the number of physicians with the training and required skill to perform these procedures as well as fear of litigation compounds, the problem of declining instrumental use even further (3),(8).

The rate of forceps should be less than vacuum roughly by half as complications are less relatively with vacuum and it needs less qualified expert than forceps. A meta-analysis of studies comparing the use of vacuum and forceps has suggested that vacuum delivery is a method of choice for instrumented delivery (8).

Although the trend in the OVD rate seen in this study is similar to what has been worldwide, it is against what is recommended in resource-limited settings, but since the study is conducted in one of the tertiary centers in Ethiopia handling most complicated pregnancies, interventions like cesarean delivery tend to be higher.

The indications for OVD are either fetal or maternal. In our study, the most frequent indication was suspected fetal distress accounting (see Table 3) for 48% of all OVD, which was similar to the result seen in 2000GC (14) 60%, although there was a decline in its percentage. Compared to the study in 2000GC, OVD for the prolonged the 2^nd^ stage increased from 19% to 34.2%, shortening of 2^nd^ stage for maternal cardiac disease, which was not reported in the 2001 study, accounted for 11.9%. The OVD for HTN was similar to 8% in 2000GC vs. 5.6% in our study, but a decline in previous CS scar as indication 4% in 2000GC vs. 0.4% in this study. Different professional societies propose different indications for the use of OVDs to their members. The practitioner must have a clear understanding of the purpose of the OVD in each particular case. This study showed that the operative vaginal delivery rate has declined from 15.8% to 9.9% over the five-year study period. The use of VAD has especially decreased compared to that of FAD. The proportion of instrument use has been shown to shift from vacuum to forceps. This could be due to the availability of instruments and other factors not assessed, but it will result in the reduction of skilled professionals that can operate, which further will decline the rate and use of instruments. Forceps are the more frequently used instruments in all listed indications in both primipara and multiparas.

This study shows that the rate of operative vaginal delivery has declined. The use of vacuum-assisted delivery has especially decreased compared to that of forceps-assisted delivery.

The trends in cesarean delivery over the five study periods (shares) were shown to have net increasing rates from 25.4% to 33.8%. The reasons for the decline in the use of instruments should be identified and measures should be taken to improve the knowledge and skill of operators and the availability of instruments. There is an urgent need to reverse the declining trend of the use of operative vaginal delivery procedures by re-introducing them into the modern obstetric curriculum, especially in resource-constrained settings like ours.
